# Horticultural, systems-engineering and economic evaluations of short-term plant storage techniques as a labor management tool for vegetable grafting nurseries

**DOI:** 10.1371/journal.pone.0170614

**Published:** 2017-02-09

**Authors:** Chieri Kubota, Chao Meng, Young-Jun Son, Myles Lewis, Hans Spalholz, Russell Tronstad

**Affiliations:** 1 School of Plant Sciences, The University of Arizona, Tucson, Arizona, United States of America; 2 Department of Systems and Industrial Engineering, The University of Arizona, Tucson, Arizona, United States of America; 3 Department of Agricultural & Resource Economics, The University of Arizona, Tucson, Arizona, United States of America; United States Department of Agriculture, UNITED STATES

## Abstract

This transdisciplinary study has a three-fold systems approach in evaluating a horticultural technology: 1) horticultural evaluations, 2) economic and resource analyses, and 3) systems engineering analyses, using low temperature storage as an example technology. Vegetable grafting is a technique to produce value-added seedlings but requires labor intensive nursery operations. Low temperature storage of seedlings for a short period of time can reduce peak production, but has not been evaluated at the extent demonstrated in this paper. Seedlings of 22 genotypes of Cucurbitaceae (cucurbit family) and Solanaceae (nightshade family) were evaluated for storability under selected temperatures and photosynthetic photon flux. Storability of Cucurbitaceous seedlings varied between 2 to 4 weeks at 12°C and 13 μmol m^-2^ s^-1^. Solanaceous seedlings were generally storable for 4 weeks at 12°C and 13 μmol m^-2^ s^-1^, but tomato seedlings could be stored for 4 weeks at 10°C and 5 μmol m^-2^ s^-1^. Capital and weekly operational costs of a low temperature storage system with a design that meets environmental requirements were estimated as $671 to $708 per m^2^ footprint and $0.79 to $2.21 per m^2^ footprint per week, respectively. Electricity costs per plant was less than 0.1 cents for 2 to 4 weeks of storage. Using a schedule-optimization heuristic and a logistics simulator previously developed for grafting nursery operations, six production scenarios consisting of two crops (tomato or watermelon) and three production peak patterns were examined to evaluate the impact of including low temperature storage. While the overall average costs of grafting labor were not significantly different, maximum labor demand and grafting labor cost during the peak production week were reduced by 31% to 50% and 14% to 30% by using storage, respectively. Therefore, low temperature storage can be an effective means to address the issue of labor management in grafting nurseries.

## Introduction

Vegetable grafting is a relatively new technology in North America, following many years of success by Asian and European countries which have been integrating grafting as a pest management practice in vegetable crop production [1–4]. By selecting matching rootstocks for their fruit producing vegetable cultivars, the technology allows growers to acquire disease resistances and abiotic environmental tolerance as well as greater overall plant vigor and yield. In European countries such as Italy, Spain, and The Netherlands, the technology has been considered essential for certain cropping systems such as greenhouse tomato (*Solanum lycopersicum*) production (for increasing yield) and watermelon (*Citrullus lanatus*) production under protected cultivation with limited or no land rotation (for controlling fusarium wilt disease). The number of grafted vegetable plants used worldwide appear to be increasing every year as a result of limited options for controlling soilborne diseases and pests with increasing concern for the environment and sustainability of vegetable production. Key issues that prevent wider adoption of grafting in North America, especially the U.S., include high prices of grafted seedlings (e.g., 40 to 90 cents per plant, excluding seed costs) associated with relatively high labor costs (40% to 60% of total costs in a manual grafting operation; [5]). A typical large farm operation in the U.S. would require a large nursery to provide seedlings during a relatively narrow, seasonal planting window, yet arranging and training a large number of required laborers for the grafting operation to achieve high success rates is challenging. As a consequence, the current U.S. vegetable grafting market is primarily for greenhouse growers and home gardeners, both of which can justify the relatively high price of grafted seedlings. The planting season is also spread out over a relatively longer period, especially for greenhouse operations. Automated grafting has been developed over the past 20 years, but the use of automation is still experimental in North American grafting nurseries, due to high capital costs and, for some cases, high material costs [5].

Plant growth rate is largely affected by temperature. The short-term low temperature storage technique for plant seedlings has been studied as an alternative strategy to mitigate peak labor demands, by distributing labor inputs for a narrow shipping window with a large peak in the amount of plants needing to be shipped. Nevertheless, despite the intensive study on optimum environmental conditions during the storage and storability of different species [6–10], the use of low temperature storage in vegetable nurseries has been limited. One reason for the underutilized status may be that the technology has never been evaluated for various aspects critical to nursery operations. First, nursery growers need to know more horticultural information regarding optimum conditions to store seedlings and their cultivar specific storability (i.e., practical length of storage period without causing negative impact to post-storage growth and development). Grafting involves two different cultivars (genotypes) selected for their phenotypes (such as vigor and disease resistances) as well as grafting compatibility. Therefore, a systematic approach is necessary to evaluate the storability of different scions, rootstocks, and their combinations. Second, engineering design requirements and costs for seedling storage facilities need to be quantified to better understand additional capital requirements. Third, the actual impact of storage in nursery logistics and possible labor savings need to be evaluated.

Logistics simulation has been shown as an effective approach applied to supply chain analyses and resource input optimization in the field of systems engineering. Simulation can incorporate discrete events (e.g., variable product demands) and ‘randomness’ of some key variables (e.g., probability of human errors in manufacturing operations) to represent the real situations. By addressing the system operations as the whole, logistic simulations enable virtually evaluating various ‘what-if scenarios’ not possible in the real systems [11]. Multiple successful applications of such simulations have been reported in the literature, such as evaluating new technologies for a logistics facility in transportation [12], health information system [13], urban highway reconstruction [14], and human cognitive workloads [15].

In this study, we have utilized a systems approach to evaluate this innovative technology of low temperature seedling storage using the following three aspects; 1) horticultural technology evaluations, 2) economic and resource analyses and finally 3) systems engineering analyses considering outcomes of the first two approaches. Horticultural technology evaluations were conducted to find the storability of selected Cucurbitaceous and Solanaceous species and cultivars typically used as scions and rootstocks in vegetable grafting at selected low temperatures. Economic and resource analyses were conducted to estimate the theoretical capital and operational costs required to introduce low temperature seedlings. The resulting information regarding the recommended storage temperature, storability of seedlings, as well as the costs was applied in the systems engineering analyses to find the impact of the low temperature storage technique to reduce overall production costs. This is the first report introducing these multi-dimensional analyses for evaluating horticultural technologies.

## Materials and methods

### Scion and rootstock plant responses to low temperatures

#### Seedlings and growing conditions

Commercially available 12 scion and 10 rootstock cultivars were selected for use in this experiment to evaluate the performance of seedlings in low temperature storage conditions. Information about each genotype such as species, common name, and scion or rootstock designation are shown in Table 1.

**Table 1:** Cucurbitaceous and Solanaceous genotypes evaluated in low temperature storage.

Common name	Species	Cultivars	Use	Days grown^Z^
Cucurbitaceous seedlings				
Cucumber	*Cucumis sativus*	Cumlaude	Scion	16
		Rembrandt	Scion	16
Muskmelon	*C*. *melo*	DRO-5018	Rootstock	24
		Honey Brew	Scion	24
		Olympic Gold	Scion	24
Watermelon	*Citrullus lanatus*	Sweet Harmony	Scion	26
		Tri-X-313	Scion	26
Bottlegourd	*Lagenaria siceraria*	Emphasis	Rootstock	16
		Macis	Rootstock	16
Interspecific squash	*Cucurbita maxima x C*. *moschata*	Strong Tosa	Rootstock	12
		Tetsukabuto	Rootstock	12
Solanaceous seedlings				
Tomato	*Solanum lycopersicum*	Aloha	Rootstock	23
		Conchita	Scion	17
		Durinta	Scion	23
Interspecific tomato	*S*. *lycopercsicum* × *S*. *habrochaites*	Maxifort	Rootstock	23
Eggplant	*S*. *melongena*	Black Bell	Scion	27
		Black Shine	Scion	27
Interspecific eggplant	*S*. *melongena* × unknown wildtype	Red Scorpion	Rootstock	24
Torvum	*S*. *torvum*	TI-216	Rootstock	30
Pepper	*Capsicum annuum*	Double Up	Scion	25
		Red Bull	Scion	25
		TI-135	Rootstock	25

^Z^ Each cultivar’s seedlings were grown for specific days in a greenhouse to reach typical grafting stages (early and late first true leaf stage for Cucurbitaceous scions and rootstocks, respectively; early second or third true leaf stage for Solanaceous scions and rootstocks).

Seeds were germinated in a temperature controlled chamber and grown for 12 to 30 days in a greenhouse (Table 1) located in Tucson, Arizona (between January and July, 2012; examining 2 to 3 cultivars at a time). The number of days to grow were decided so that seedlings reached their typical grafting stage (i.e., the beginning of first true leaf stage for Cucurbitaceous seedlings and the beginning of second or third true leaf for Solanaceous seedlings). Plastic 1020-type seedling trays (72 and 98 cells/tray for Cucurbitaceous and Solanaceous seedlings, respectively) were filled with a commercial substrate (Sunshine Mix #3; Sun Gro Horticulture, Bellevue, WA) and sub-irrigated with water until cotyledons emerge, which was then replaced by a multi-crop hydroponic solution developed at the University of Arizona (Jensen and Rorabaugh, unpublished) consisting of mg/L (ppm) 90 N, 47 P, 144 K, 160 Ca, 60 Mg, 114 S, 88 Cl, 0.34 B, 0.55 Mn, 0.05 Cu, 0.05 Mo, and 0.33 Zn.

The greenhouse used for this study was covered with twin-wall acrylic panels, and equipped with a pad-and-fan evaporative cooling system and an over-head air heating system. In addition, a high pressure fogging system was used to maintain the greenhouse relative humidity above 55% during the daytime. Greenhouse air temperature was set at 24°C during the day and 12°C during the night. Seedlings were placed on heating mats and covered with a tent made of nonwoven cloth to maintain a night time substrate temperature of 20°C, unless this temperature was met by ambient greenhouse conditions.

#### Storage conditions and analyses

A total of 30 seedlings were selected randomly from the tray for each cultivar (a total of 690 seedlings for each temperature) at their typical grafting stage, and placed in a new seedling tray (the same 72 or 98 cell counts as stated earlier) inside a semi-transparent plastic storage box (volume 52.9 liter) in a walk-in chamber (floor area: 14.8 m^2^) controlled at 10°C (Solanaceous seedlings) or 12°C (Cucurbitaceous and Solanaceous seedlings) for up to 4 weeks. Each storage box was covered by a single layer of clear polyethylene plastic film for humidification and contained a small dish holding 100 g of potassium permanganate impregnated porous media (GC PPA8; Filter Innovations, Toronto, ON, Canada) as an ethylene absorbent. After 2 weeks it was replaced with fresh potassium permanganate impregnated porous media. Cool white fluorescent lamps (40 W F40T12/CW PLUS/ALTO; Philips Lighting, The Netherlands) were used as the light source. In each shelf, one fluorescent lamp was mounted at 80 cm above the shelf surface to deliver an average photosynthetic photon flux (PPF) of 5 ± 0.2 (s.d.) and 13 ± 0.75 (s.d.) μmol m^-2^ s^-1^ at 10 and 12°C respectively, over the plant canopy inside the container. The PPF was adjusted using black plastic-mesh shade cloth in this experiment (see the photo in S1 File). These PPF set-points were selected to achieve a near light compensation point for these species, based on the previous studies [9, 16]. Plants were irrigated well prior to the storage. During the storage experiment, irrigation was not necessary as humidity levels were very high inside storage boxes and substrates were moist for 4 weeks. Storage box temperatures were monitored by using dataloggers (Hobo Pro Series; Onset, Pocasset, MA), using one datalogger for each chamber.

After 4 weeks in storage, seedlings in the same multi-cell trays were placed back in the greenhouse to assess seedling quality using the same visual rating for additional 2 weeks. Stored seedlings required a 2-day post-storage acclimation. Acclimation conditions consisted of 80% shade cloth on the first day and 50% on the second day. After 1 week of post-storage in the greenhouse, seedlings were transplanted into 3-inch pots filled with the same commercial substrate mix described earlier. The plants were sub-irrigated with the same nutrient solution as described earlier when necessary to maintain ample substrate moisture. Every third sub-irrigation substituted water for the nutrient solution to prevent salt accumulation. Plants remained in the greenhouse for 2 weeks.

During the 4-week storage and the 2-week post storage in the greenhouse, visual scoring was employed to assess overall seedling quality and condition. Visual scores were taken of each sample according to Justus and Kubota [9] as shown in Table 2. All visual scoring was performed by the same individual. Due to the difficulty to separate the substrate materials from the roots, our assessment was limited to overall shoot quality and conditions, not including root conditions. Hierarchical cluster analysis (Ward’s method) was applied to evaluate how the groups of scions and rootstocks respond to storage using JMP software (version 9.0; SAS Institute, Cary, NC).

**Table 2:** Visual quality score criteria applied in- and post-storage evaluations.

Visual Score	Plant Condition	Marketable
1	Dead plant or scion with no living leaves or apical meristem	No
2	Wilting, leaf tip senescence; with chlorosis or necrosis on the cotyledons and true leaves	No
3	Chlorosis or yellowing of cotyledons and minor symptoms to upper leaf canopy including leaf tip and leaf edge senescence	Yes
4	Healthy plant with a visual decrease in chlorophyll; lighter in color	Yes
5	Plants are green and overall healthy, ideal seedlings with no visual decrease in quality that are completely healthy	Yes

Adopted from Justus and Kubota [9].

### Estimation of storage capital and electricity costs of operation

The capital and operating costs for low temperature storage were estimated theoretically in a similar way that Lewis et al. [5] did for a grafting healing chamber, the same type of walk-in structure having lighting and cooling systems as the low temperature storage chamber in this study. The low temperature storage chamber size used as this design base for the cost analysis was 7.6 m (W) x 7.6 m (D) x 3.7 m (H) having a 214 m^3^ volume. The chamber was a standard insulated modular structure with walls made of insulated panels (10 cm thickness) to minimize energy consumption. We assumed that these chambers were placed inside a warehouse building protected from the outside climate. Inside the chamber, there were two shelving units equipped with lighting systems. Each shelving unit had 5 layers each with a size of 7.6 m x 3.4 m x 0.6 m. Maximum storage capacity of the chamber was 960 trays (0.23 m^2^ tray size). Planting density over tray surface employed in this cost estimation was 882 plants m^-2^ for tomato and 565 plants m^-2^ for watermelon.

The cost of building a modular structure chamber was quoted from a commercial source for prefabricated insulated structures (Bally Refrigerated Boxes, Morehead City, NC), and included the costs for cooling and lighting equipment which had the capacity to meet the required conditions. The number of lamps to obtain target light intensity over the plant canopy (5 or 12 μmol m^-2^ s^-1^ PPF) was estimated following the procedure used by Ohyama and Kozai [17]. Briefly, it was estimated based on the shape of each shelf (room index = 3.82), reflectance of each surface of the shelf (70%, 30%, and 10% for the upper surface, wall, and lower surface inside the shelf, respectively), and the PAR photon emission rate of luminaires (37 μmol s^-1^ per luminaire, 400–700 nm). We assumed that ordinary white fluorescent lamps (32 W, T8 type) were used in the chamber. The capital cost specific to the chamber was converted to the cost per unit size of footprint (m^2^). The cooling equipment was selected based on the total cooling load (lighting and heat exchange between inside and outside of the chamber).

The main costs of operating storage chambers are typically the electricity used for lighting and cooling. Outside conditions of the storage chamber (inside the warehouse) were assumed as 15°C and 50% RH for this analysis. Light in this chamber is used continuously (24 hours per day). The electric power consumption for cooling was estimated using the total cooling load considering lighting, heat exchange between inside and outside of the chamber, and the cooling performance of selected cooling unit (COP, coefficient of performance) available from the manufacturer (i.e., COP = 2.93 in this study). Air temperature and PPF inside the storage examined in this study were 10°C and 5 μmol m^-2^ s^-1^ for tomato seedlings or 12°C and 12 μ mol m^-2^ s^-1^ for watermelon seedlings. The relative humidity was assumed to be 60% in the chamber. All key parameters used in the cost analysis can be found in the supplemental file (S2 File).

### Systems engineering analyses–scenario based simulations

Following the horticultural evaluation and baseline costs analyses, we conducted systems engineering analyses based on the selected scenarios. Our goal was to find the benefit of using low temperature storage in terms of potential labor cost savings. This part of the study was also focused on two major species grafted worldwide, tomato and watermelon. We used a previously developed simulator [18] using SimCad (ver. 12.2; Create ASoft, Naperville, IL) consisting of a user interface for defining scenario parameters as simulation inputs, heuristics for optimizing weekly grafting schedule considering low temperature storage, and a data-driven grafting propagation simulator for evaluating the performance of grafting propagation.

The user interface was designed in Microsoft Excel (ver. 15.0), and provided three categories of nursery operation-related parameters, such as facility, production and labor via various spreadsheets [18]. In this study, we defined three production peak patterns with different demand peaks for the two species (tomato and watermelon) at the estimated storage chamber electricity consumption rates, grafting labor (both skilled and unskilled) capacity, and salary rates via the user interface.

Embedded in the user interface, a heuristic was proposed to determine the optimal grafting schedule by spreading the weekly grafting quantities during peak season to pre-peak weeks considering the capacity of low temperature storage. First, the heuristic identifies the number of peaks and the corresponding weekly shipment quantities, given the production peak pattern defined by the scenario. According to the scale of the weekly shipment requirement, the heuristic then decomposes the quantity of each week into blocks of the same size (e.g. 10 seedling trays per block), and moves the blocks forward to generate the grafting schedule under the constraint of the maximum storage weeks allowed for each crop type. In this study, the heuristic selected the optimal grafting schedule based on two objectives. The primary objective was to minimize grafting peak size, namely, the maximum weekly grafting quantity during the given production period to simulate. If more than one schedule candidate had the same maximum weekly grafting quantity, the heuristic then selected the one that minimizes the variance of weekly grafting quantities for the planning horizon (the second objective). In the instance that more than one schedule candidate happened to achieve the same performance with respect to those two objectives, the heuristic randomly selected one as the output.

**Fig 1:**
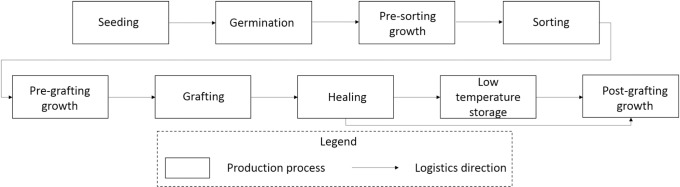
Processes modeled in the logistics simulation. Grafted seedling production involves multiple processes including seeding, germination in a temperature controlled chamber or greenhouse, pre-sorting growth in greenhouse, sorting (a process to create uniform seedling stands), pre-grafting growth (or post sorting growth) in greenhouse, grafting, healing in a healing chamber (a propagation stage to complete the graft unions critical in grafting nursery operation), and post-grafting growth stages to finish the seedlings in the greenhouse. In this study, we examined the impact of introducing low temperature storage (after healing) in production scheduling, grafting labor input and costs.

The simulator used in this study [18] simulates logistics of the whole grafting nursery operation of seeding, germination, pre-sorting growth, sorting (a process to create uniform seedling stands), pre-grafting growth (or post sorting growth), grafting, healing (a propagation stage to complete the graft unions critical in grafting nursery operation), low temperature storage and post-grafting growth stages (Fig 1). In this study, a standard duration from seeding to grafting was 3 weeks for tomato (i.e., second true leaf stage) and 2 weeks for watermelon (first true leaf stage), followed by one week of healing and 2 weeks of finishing/hardening to shipment (3 to 5 true-leaf developmental stage). These durations varied stochastically within ±21% to ±23% standard deviations considering possible changes in plant growth caused by various factors such as greenhouse climate conditions caused by unexpected weather conditions (e.g., overcast day). We have also considered variability caused by loss of seedlings (±5%) as well as grafting speed (±10%). In the simulation, according to the optimized grafting schedule described above, healed grafts were transported to either the low temperature storage or the greenhouse for post-grafting growth. Simulation outputs included the required space and operating costs of low temperature storage, grafting labor (skilled and unskilled) required and grafting labor costs per plant for each week of simulation. Since the simulation is dynamic, the required size of low temperature storage was determined based on the maximum trays in storage. In addition, the number of skilled grafting workers required was determined based on the minimum weekly grafting production quantity. Correspondingly, the number of unskilled grafting workers (seasonal or migrant workers) for each week was determined to supplement the grafting capacity needed to fulfill the scheduled grafting production quantity. Skilled grafting worker wage was considered as 50% greater than those of unskilled grafting workers ($7.80 per hour minimum wage). Seasonal workers are locally available temporary workers (up to 50 workers per week at the minimum wages plus 25% fringe benefits) and migrant workers are those who need arrangements for temporary housing and other expenses ($17.50 per day) in addition to the hourly wages and one-time transportation cost from the origin ($500 in this study). In this simulation, employing migrant workers was considered for the week requiring more than 50 additional workers. Wage rate and necessary costs (overhead, meals, housing compensations) for seasonal and migrant workers were considered based on the available resources and interviews through relevant government agencies.

**Fig 2:**
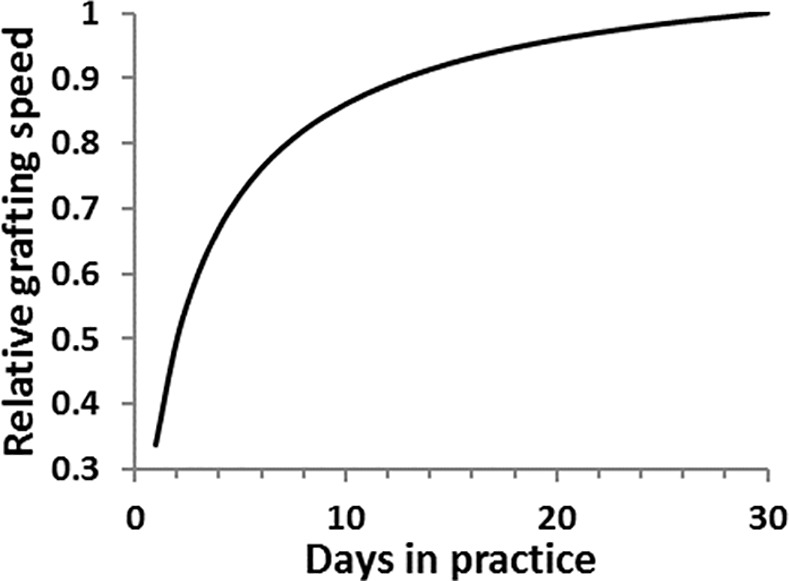
Workers learning curves employed in this simulation. Relative speed of grafting is shown using the equation, *T* = *X* + *N·P*^*c*^, where *T* is the time to graft one seedling, *P* is the number of days for grafting practice, and *X*, *N*, *c* are constants defining the shape of learning curve (S3 File).

In this analysis, we incorporated the learning curve (Fig 2) proposed in Snoddy [19] to represent the learning process of unskilled grafting workers in terms of grafting speed. The initial grafting speed for tomato and watermelon were selected as 36 and 64 seconds per plant (100 and 56 plants per hour), respectively, and the maximum grafting speed that unskilled grafting workers can achieve were 12 and 24 seconds per plant (300 and 150 plants per hour) for tomato and watermelon, respectively. Based on the data provided by a major grafting propagator located in North America, the learning period was set to be 3 weeks. Skilled workers’ grafting speeds were kept at the maximum level of 12 and 24 seconds per plant for tomato and watermelon, respectively.

Due to the randomness embedded in the simulator, five replicated simulation runs were conducted for each scenario, followed by T-test. The parameters of grafting propagation used in this study were set based on the Tucson, Arizona area (S3 File).

## Results and discussion

### Storability of different scion and rootstocks used for grafting

Table 3 shows the visual scores of 11 Cucurbitaceous scion and rootstock seedlings over the 4-week storage period at 12°C followed by 2-weeks of post-storage growth in the greenhouse. The cluster analysis classified interspecific hybrid squash rootstocks (Strong Tosa and Testukabuto), cucumber scion cultivars (Rembrandt and Cumlaude) as well as one muskmelon rootstock (DRO-5018) as a relatively tolerant group to the 12°C low temperature conditions. The second group included both bottle gourd rootstocks (Emphasis and Macis). These second group genotypes had a visual score below 4.0 after 21 days in storage, mainly due to leaf yellowing, but they recovered their rating to 5.0 during the 2 weeks of post-storage growth. The genotypes we found relatively sensitive to the 12°C storage included muskmelon and watermelon scion cultivars (Honey Brew, Olympic Gold, Sweet Harmony, and Tri-X-313), showing overall leaf yellowing and/or necrosis that progressed over time. Among this group, ‘Sweet Harmony’ and ‘Tri-X-313’ watermelon scion seedlings as well as ‘Olympic Gold’ muskmelon seedlings did not recover their visual quality scores above a marketable level of 3.0. These scion cultivars are typically grafted onto an interspecific hybrid squash rootstock, which we found more tolerant in the low temperature of 12°C. In fact, Justus and Kubota [9] found that low-temperature sensitive ‘Olympic Gold’ muskmelon seedlings were stored up to 4 weeks without causing significant negative effects on post-storage growth and development when grafted onto the ‘Testukabuto’ interspecific hybrid squash rootstock. This result demonstrated the acquisition of low temperature tolerance by grafting. Similarly, our separately-conducted study showed that the storability of ‘Tri-X-313’ watermelon seedlings were improved by grafting either on the ‘Strong Tosa’ interspecific hybrid squash rootstock or ‘Emphasis’ bottle gourd rootstock [20]. Low temperature tolerance is in fact one of the physiological changes observed in grafted cucurbit plants and a wide range of physiological studies have been conducted [21–26]. For example, Tachibana [22, 23] and Ahn et al. [24] showed improved ion uptake at low temperature for grafted plants. Lee et al. [25] found that low temperature resistance was associated with high hydraulic conductivity maintained at sub-optimal temperature. Zhang et al. [26] also reported that ‘root-to-shoot communication’ in cucumber under low temperature was dominated by water status reducing photosynthetic activities rather than ABA signal transduction under abiotic stress. These observations support the high tolerance to low temperatures of certain rootstocks and therefore of the grafted plants at low temperatures that otherwise cause low-temperature induced wilting in sensitive scion seedlings.

**Table 3:** Visual scoring of Cucurbitaceous seedlings during the 4-week storage at 12°C (Days 1 to 28) followed by 2-week post-storage in greenhouse (Days 31 and 42).

Cultivars	Common Name	12°C Storage^Z^					Post Storage^Z^		Average^Y^ & cluster analysis
		Day 1	Day 7	Day 14	Day 21	Day 28	Day 31	Day 42	
Strong Tosa	Squash (R)	5.0±0.0	5.0±0.0	5.0±0.0	5.0±0.0	5.0±0.0	5.0±0.0	5.0±0.0	5.0 A
Tetsukabuto	Squash (R)	5.0±0.0	5.0±0.0	5.0±0.0	4.9±0.1	4.8±0.1	4.9±0.1	5.0±0.0	4.9 A
Rembrandt	Cucumber (S)	5.0±0.0	5.0±0.0	5.0±0.0	4.8±0.1	4.6±0.1	4.9±0.1	5.0±0.0	4.9 A
Cumlaude	Cucumber (S)	5.0±0.0	5.0±0.0	5.0±0.0	4.6±0.1	3.5±0.2	4.5±0.1	5.0±0.0	4.7 A
DRO-5018	Muskmelon (R)	5.0±0.0	5.0±0.0	4.9±0.1	4.0±0.1	3.9±0.1	3.7±0.1	4.4±0.2	4.4 A
Emphasis	Bottle gourd (R)	5.0±0.0	5.0±0.0	4.0±0.0	3.9±0.1	3.8±0.1	4.0±0.0	5.0±0.0	4.4 B
Macis	Bottle gourd (R)	5.0±0.0	5.0±0.0	4.0±0.0	3.7±0.2	3.7±0.2	4.0±0.0	5.0±0.0	4.3 B
Honey Brew	Muskmelon (S)	5.0±0.0	5.0±0.0	4.3±0.1	3.9±0.1	3.3±0.1	2.9±0.2	3.4±0.3	4.0 C
Tri-X-313	Watermelon (S)	5.0±0.0	5.0±0.0	4.5±0.1	3.9±0.2	3.5±0.2	1.9±0.1	2.7±0.2	3.8 C
Olympic Gold	Muskmelon (S)	5.0±0.0	5.0±0.0	4.1±0.1	3.3±0.2	3.0±0.2	2.2±0.2	2.7±0.3	3.6 C
Sweet Harmony	Watermelon (S)	5.0±0.0	5.0±0.0	4.0±0.0	2.7±0.3	2.5±0.2	1.1±0.1	1.1±0.1	3.0 D

^Z^ Averages and standard error (n = 15) for each genotype of each observed day.

^Y^ Average of visual scores over 6 weeks. Hierarchical cluster analysis was applied over the 6-week trend in change of visual scores using Ward’s method.

S = Scion, R = Rootstock. See Table 2 for the criteria of scoring.

The most sensitive cultivar to the low temperature among the 11 genotypes examined in the present study was ‘Sweet Harmony’ watermelon. The seedling’s visual score dropped below 3.0 after 3 weeks in storage, and did not recover during the post storage growth in the greenhouse. Based on this, we decided to use watermelon in the following analyses and considered that grafted watermelon seedlings can be stored for only up to 2 weeks under 12°C without causing negative influence on post-storage growth and development. This storability of grafted watermelon plants was later confirmed in a separate experiment [20]. Table 4 shows the visual scores of 11 Solanaceous seedlings for the 4-week storage at 10 or 12°C followed by 2-week post-storage growth period in the greenhouse. At 10°C, the seedlings of the intra-specific hybrid tomato rootstock (Aloha) and one of tomato scions (Durinta) maintained high visual scores throughout the experiment. Eggplant scion cultivars (Black Bell and Black Shine) showed the greatest decline in visual score to 3.0 due to leaf yellowing after 28 days in storage, but recovered their visual rating to 5.0 after 2 weeks of post-storage growth. All other genotypes showed intermediate response and recovered their rating to 5.0 during the 2-week post storage growth period in the greenhouse.

**Table 4:** Visual scoring of Solanaceous seedlings during the 4-week storage at 10 or 12°C (Days 1 to 28) followed by 2-week post-storage in greenhouse (Days 31 and 42).

Cultivars	Common Name	10 or 12°C Storage^Z^					Post Storage^Z^		Average^Y^ & cluster analysis
		Day 1	Day 7	Day 14	Day 21	Day 28	Day 31	Day 42	
Storage at 10°C									
Aloha	Tomato (R)	5.0±0.0	5.0±0.0	5.0±0.0	5.0±0.0	5.0±0.0	5.0±0.0	5.0±0.0	5.0 A
Durinta	Tomato (S)	5.0±0.0	5.0±0.0	5.0±0.0	5.0±0.0	5.0±0.0	5.0±0.0	5.0±0.0	5.0 A
Conchita	Tomato (S)	5.0±0.0	5.0±0.0	5.0±0.0	5.0±0.0	4.0±0.0	5.0±0.0	5.0±0.0	4.9 B
TI-135	Pepper (R)	5.0±0.0	5.0±0.0	5.0±0.0	5.0±0.0	4.0±0.0	4.9±0.1	5.0±0.0	4.9 B
Red Bull	Pepper (S)	5.0±0.0	5.0±0.0	5.0±0.0	5.0±0.0	3.8±0.1	5.0±0.0	5.0±0.0	4.9 B
TI-216	Torvum (R)	5.0±0.0	5.0±0.0	5.0±0.0	5.0±0.0	3.9±0.1	5.0±0.0	5.0±0.0	4.8 B
Double Up	Pepper (S)	5.0±0.0	5.0±0.0	5.0±0.0	5.0±0.0	3.7±0.1	5.0±0.0	5.0±0.0	4.8 B
Red Scorpion	Eggplant (R)	5.0±0.0	5.0±0.0	5.0±0.0	5.0±0.0	3.4±0.1	5.0±0.0	5.0±0.0	4.8 B
Maxifort	Tomato (R)	5.0±0.0	5.0±0.0	4.7±0.1	4.6±0.1	3.8±0.1	3.9±0.1	5.0±0.0	4.7 C
Black Shine	Eggplant (S)	5.0±0.0	5.0±0.0	5.0±0.0	3.8±0.1	3.0±0.0	3.0±0.0	5.0±0.0	4.3 D
Black Bell	Eggplant (S)	5.0±0.0	5.0±0.0	5.0±0.0	3.4±0.1	3.0±0.0	3.0±0.1	5.0±0.0	4.2 D
Storage at 12°C									
TI-135	Pepper (R)	5.0±0.0	5.0±0.0	5.0±0.0	5.0±0.0	5.0±0.0	5.0±0.0	5.0±0.0	5.0 a
Red Scorpion	Eggplant (R)	5.0±0.0	5.0±0.0	5.0±0.0	4.9±0.1	4.0±0.0	4.7±0.1	5.0±0.0	4.8 a
Conchita	Tomato (S)	5.0±0.0	5.0±0.0	4.9±0.1	4.1±0.1	4.1±0.1	4.8±0.1	5.0±0.0	4.7 a
Double Up	Pepper (S)	5.0±0.0	5.0±0.0	4.5±0.1	4.4±0.1	4.2±0.1	4.6±0.1	5.0±0.0	4.7 a
Red Bull	Pepper (S)	5.0±0.0	5.0±0.0	4.7±0.1	4.3±0.1	4.0±0.0	4.7±0.1	5.0±0.0	4.7 a
Durinta	Tomato (S)	5.0±0.0	5.0±0.0	5.0±0.0	5.0±0.0	4.0±0.0	4.0±0.0	5.0±0.0	4.7 b
Maxifort	Tomato (R)	5.0±0.0	5.0±0.0	4.9±0.1	4.9±0.1	4.0±0.0	3.9±0.1	5.0±0.0	4.7 b
Aloha	Tomato (R)	5.0±0.0	5.0±0.0	5.0±0.0	5.0±0.0	4.0±0.0	4.0±0.0	5.0±0.0	4.7 b
TI-216	Torvum (R)	5.0±0.0	5.0±0.0	4.8±0.1	3.7±0.2	3.5±0.1	4.0±0.0	5.0±0.0	4.4 c
Black Bell	Eggplant (S)	5.0±0.0	5.0±0.0	4.7±0.1	3.2±0.1	3.0±0.0	4.1±0.1	5.0±0.0	4.3 c
Black Shine	Eggplant (S)	5.0±0.0	5.0±0.0	4.0±0.0	3.0±0.0	3.0±0.0	4.0±0.0	5.0±0.0	4.1 d

^Z^ Averages and standard error (n = 25 for 10°C and n = 15 for 12°C) for each genotype of each observed day.

^Y^ Average of visual scores over 6 weeks. Hierarchical cluster analysis was applied over the trend in change of visual scores using Ward’s method.

S = Scion, R = Rootstock. See Table 2 for the criteria of scoring.

When stored at 12°C, most genotypes except eggplant scion cultivars (Black Bell and Black Shine) and Torvum rootstock ‘TI-216’ maintained their visual rating above 4.0 during the 4-week storage. ‘Aloha’ tomato rootstock and ‘Durinta’ tomato scion cultivar were placed in the second tolerant group by the cluster analysis at 12°C while they were the most tolerant group at 10°C, suggesting that 12°C is a super-optimum temperature that shortened their storability. Of interest ‘Red Scorpion’ interspecific hybrid eggplant rootstock exhibited high tolerance to the low temperature of 12°C compared with eggplant scion cultivars. To our knowledge, this interspecific eggplant is a hybrid between *S*. *melongena* and an undisclosed wild type (personal communication with Takii Seeds, Kyoto, Japan). Grafting eggplant using this rootstock could possibly convey the low temperature tolerance, as demonstrated in Cucurbitaceous plants [9]. In contrast, Torvum rootstock ‘TI216’ is widely used for grafting eggplant and is considered as sensitive to 12°C low temperature as eggplant scion cultivars. Therefore, Eggplant scion grafted on to Torvum rootstock may be the most sensitive combination of scion/rootstock to low temperature among the Solanaceous genotypes examined in this study. Other than these specific scions, rootstock and their possible combinations, it can be concluded that Solanaceous seedlings (and potentially their grafts) are highly storable up to 4 weeks at 10 to 12°C under 5 to 13 μmol m^-2^ s^-1^ lighting. In the following cost analyses and logistics simulation, we considered that grafted tomato seedlings could be stored for 4 weeks under 10°C.

Light intensity suitable for low temperature storage is selected using the seedling light compensation point at the temperature as the reference point. The light compensation point generally decreases with lower temperatures. Kubota et al. [7] demonstrated that broccoli seedlings’ light compensation point reduced from 5 μmol m^-2^ s^-1^ to 2 μmol m^-2^ s^-1^ PPF as temperature was decreased from 10°C to 5°C. While the reduction of air temperature may be considered as leading to a potential increase in cooling costs, the decrease in PPF requirement at a lower temperature also benefits the operational electricity costs, as the lights in storage are the major source of cooling load inside a well-insulated storage chamber. Fujiwara et al. [27] showed that light compensation point changes over time during the storage as seedlings are acclimated to the low light environment. This aspect may be considered in selecting light intensity for storage after further investigation is made for specific genotypes (e.g., cultivars) when applying storage technique.

### Capital and electricity costs of low temperature storage

Total capital costs or the investment needed to introduce the capacity of low temperature storage in an existing nursery operation was estimated as $671.16 and $707.88 per square meter of storage structural footprint for tomato and watermelon, respectively (Table 5). The estimated capital requirement was 5.5% greater for watermelon than for tomato, which is attributed to the difference in lighting requirements between these two species. Light intensity over the shelves inside the low temperature storage was selected as 12 μmol m^-2^ s^-1^ for watermelon, 2.4 times greater than the level for tomato (5 μmol m^-2^ s^-1^) due to the warmer storage temperature for watermelon (12°C) compared to tomato (10°C). As expected, the highest capital related expense was the structure (a standard insulated modular structure), 75 to 79% of the total capital cost. The second largest capital item was the shelving units, followed by luminaires. Luminaire costs were relatively small compared with the total costs (4.3% for tomato and 9.2% for watermelon). Selecting the optimum light intensity is crucial in seedling storage [6, 8] and storage chamber design should consider acquiring the highest possible light intensity at selected temperatures. In the present analysis, the capital costs were estimated using a selected size of storage system and compatible equipment (shelving units, lamps, cooling system) as the design base to compute unit-area based costs. The size of chamber would affect the capital cost. Further analyses need to be done with actual sizes used for individual business situations.

**Table 5:** Capital and electricity costs ($US) for a low temperature storage chamber.

Cost items	Storage chamber settings (temperature/PPF^Z^)	
	10°C/5 μmol m^-2^ s^-1^ (tomato)	12°C/12 μmol m^-2^ s^-1^ (watermelon)
Capital input per m^2^ footprint^Y^		
Structure	$533.47	$533.47
Shelving units	$84.61	$84.61
Luminaires	$28.56	$65.28
Chiller unit	$24.53	$24.53
Total capital input	$671.16	$707.88
Operation costs		
Electricity per m^2^ footprint per week	$0.79	$2.21
Electricity per plant per week	$0.00024	$0.00104

^Z^ Photosynthetic photon flux.

^Y^ Storage capacities of 3,307 and 2,116 plants per m^2^ footprint for tomato and watermelon, respectively.

Electricity costs per week were estimated as $0.79 m^-2^ footprint for tomato and $2.21 m^-2^ for watermelon. This difference is attributed to the differences in lighting (12 vs. 5 μmol m^-2^ s^-1^ PPF) required between the two species. Lighting could be further reduced by introducing more efficient lamps such as LEDs, but a drawback to this is higher capital costs as LEDs are 2 to 6 times more expensive per mole of PAR photon emission [28]. Despite the relatively low efficiency of fluorescent lamps considered in the present study, the storage electricity costs per plant per week was $0.00024 and $0.00104 for tomato and watermelon, negligibly small relative to all the production costs of grafted plants. For example, Lewis et al. [5] estimated total variable costs ranging between $0.089 and $0.195 per plant (excluding seed costs), depending on the grafting operation scale of the grafting nursery and technology level (automated grafting vs. manual grafting). Rivard et al. [29] reported much higher costs for small commercial nurseries located in the eastern U.S. of $0.59 to $1.25 (or $0.31 to $0.93 when excluding seed costs).

In the present study, the air temperature and relative humidity outside the low temperature chamber were selected as 15°C and 50%. Due to the limited heat exchange between inside and outside of the chamber, the effect of ambient conditions on the electric energy consumption is minimal (5.1% and 1.4% for tomato and watermelon, respectively; S2 File). Outside temperature affects the overall heat exchange of the chamber as well as COP of the cooling unit. Higher ambient temperature likely increases the electricity costs further. For example, Tucson annual average temperature is 19.9°C. At a 20°C outside temperature, due to the increased overall heat flux from outside to inside the chamber, electricity costs will increase by approximately 2 times for tomato and 2.7 times for watermelon, compared with those at 15°C. Lewis et al. [5] analyzed the grafting costs for tomato and watermelon and reported that production costs of grafted plants include 4.9 and 3.2 cents per plant for utility costs of tomato and watermelon, respectively. Compared with these costs, the additional increase in electricity estimated with low temperature storage should be acceptable (i.e., less than 0.1 cents for either 4- or 2- week storage of tomato or watermelon, respectively).

### Possible mitigation of labor input and costs by introducing low temperature storage

While the systems engineering analyses can be applied to a multitude of scenarios, six production scenarios consisting of two crops and three production peak patterns were examined to evaluate the impact of including low temperature storage in this analysis. As low temperature storage by nature is a means to mitigate peak demand (especially labor), our scenario based evaluations focused on the impact of low temperature storage on labor input and costs of the grafting process.

**Fig 3:**
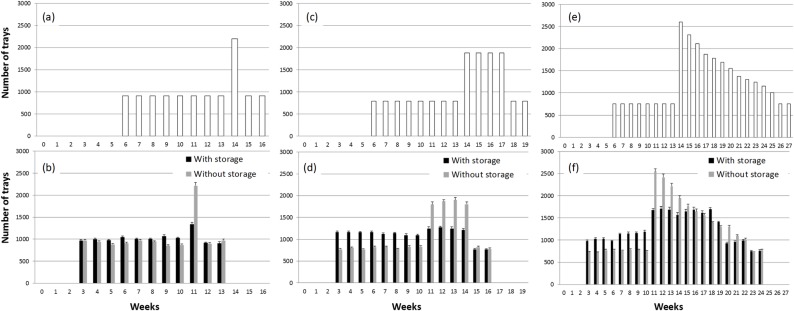
**Shipment schedules for tomato in number of grafted seedling trays per week under the selected three production scenarios (a, c, e) and number of trays that needed to be grafted (b, d, f) to meet the production schedule with and without using low temperature storage (means and margins of error (ME, *P*** **≤** **0.05) of five simulations).** Maximum storage duration was up to 4 weeks.

**Fig 4:**
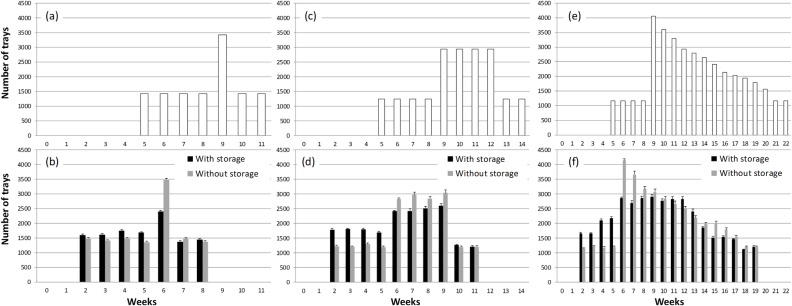
**Shipment schedules for watermelon in number of grafted seedling trays per week under the selected three production scenarios (a, c, e) and number of trays that needed to be grafted (b, d, f) to meet the production schedule with and without using low temperature storage (means and margins of error (ME, *P*** **≤** **0.05) of five simulations).** Maximum storage duration was up to 2 weeks.

The three production scenarios examined (Figs 3 and 4) were distinct in nature as; 1) single-week production peak, 2) multi-week consistent production peak, and 3) multi-week descending production peak. Our optimization heuristic found a workable schedule of grafting for each scenario with a lower peak number of trays needed to process (graft) with a relatively more even distribution over time by introducing low temperature storage, compared to those without storage (Figs 3 and 4). However, due to the relatively short storability of grafted watermelon seedlings, low temperature storage could not distribute the production for watermelon (Fig 4) as evenly as for tomato (Fig 3), in comparison to production without storage. This was especially the case for the first and second production scenarios (i.e., single and multi-week peaks). As a result, the maximum number of workers needed were reduced by 31 to 37% for watermelon, a lesser degree than for tomato (48 to 50%) by introducing low temperature storage in the first and second production peak scenarios (Table 6). In contrast, the reduction in peak labor was almost the same (47 to 48%) for both crops for the 3^rd^ production peak scenario, where the production peak has a descending level over 12 weeks.

**Table 6:** Labor requirement per week (maximum, minimum and average) and peak-week and overall grafting labor costs estimated per plant as affected by introducing low temperature storage (LTS) for the selected three production scenarios (shown in Figs 3 and 4) for tomato and watermelon grafted plants.

		LTS^Z^	Weekly Max./min. labor need (persons)	Weekly average labor need^Y^ (persons ± S.D.)	Grafting labor costs during peak production week^X^ ($/plant ± S.D.)	Overall average of grafting labor costs^Y^ ($/plant ± S.D.)
Tomato	Scenario 1	Yes	27 / 15	18 ± 3.3^NS^	$0.054 ± 0.0014*	$0.049 ± 0.0024^NS^
		No	52 / 15	19 ±11	$0.063 ± 0.0016	$0.050 ± 0.0047
	Scenario 2	Yes	23 / 13	19 ± 2.9 ^NS^	$0.051 ± 0.0013*	$0.048 ± 0.0023^NS^
		No	46 / 13	20 ±11	$0.060 ± 0.0016	$0.049 ± 0.0041
	Scenario 3	Yes	36 / 13	22 ± 6.0^NS^	$0.054 ± 0.0014*	$0.047 ± 0.0037^NS^
		No	68 / 13	22 ± 13	$0.070 ± 0.0018	$0.048 ± 0.0060
Watermelon	Scenario 1	Yes	62 / 30	39 ±11^NS^	$0.11 ± 0.0019*	$0.098 ± 0.0060^NS^
		No	99 / 30	40 ±26	$0.13 ± 0.0023	$0.10 ± 0.014
	Scenario 2	Yes	59 / 26	43 ±12^NS^	$0.10 ± 0.0017*	$0.097 ± 0.0057^NS^
		No	85 / 26	44 ±23	$0.12 ± 0.0021	$0.097 ± 0.011
	Scenario 3	Yes	74 / 25	47 ± 15^NS^	$0.110 ± 0.0019*	$0.094 ± 0.0075^NS^
		No	124 / 25	48 ± 26	$0.157 ± 0.0027	$0.096 ± 0.016

^Z^ Maximum storage duration was 4 and 2 weeks for tomato and watermelon, respectively.

^Y^ Means ± standard deviation.

^NS^ Non-significantly different by T-test (*P*<0.05, n = 11, 14, 22, 7, 10, and 18 for tomato and watermelon scenarios 1, 2, and 3, respectively).

^X^ *Means significantly different by T-test at (*P* ≤ 0.05, n = 5).

When low temperature storage was introduced, weekly labor needs were also relatively stable (i.e., smaller standard deviations) compared to an operation without storage. This reduction of peak labor need is significant since total labor costs accounted for 40% to 60% of all production costs when grafted manually [5]. While overall grafting labor costs were not significantly different with and without low temperature storage, incorporation of storage significantly reduced weekly grafting labor costs during the peak for each scenario (by 14% to 30%) (Table 6 and S3 File). This was mainly due to the overall higher grafting skill level and productivity (Fig 2) of unskilled workers by employing them for a longer time period. In addition, to satisfy peak weekly demand for tomato’s 3^rd^ scenario and all watermelon’s scenarios, more costly migrant workers had to be employed in addition to seasonal local workers when operated without storage (S3 File). Reducing or eliminating migrant worker needs also contributed to lower labor costs with low temperature storage during these peak periods.

Horticultural operations, especially fruit and vegetable production as well as nursery plant production (including grafting), are labor intensive. In such industries in the U.S., labor makes up about half of the variable production expenses [30], and most manual workers contributing to the high labor cost are migrant workers arranged through the guest worker program. Calvin and Martin [30] report labor issues and labor’s status in various cases of the U.S. fresh produce industry. They note that typical solutions that industry takes include using less labor or more efficiently by applying labor aides or mechanization. Calvin and Martin [30] also cite the U.S. Department of Labor’s National Agricultural Workers Survey (NAWS) and note that most hired workers stay in the seasonal farm workforce a decade or less, and as a consequence, employers are constantly looking for new workers [31]. The constant need to train new unskilled workers is what we have observed in horticultural nurseries and obviously a drawback of skill-based operations like vegetable grafting. The low temperature storage technique examined in this study was shown as a means to improve overall labor efficiency by giving longer employment time to improve average skill levels.

In conclusion, our integrated systems approach for evaluating the technique of low temperature storage of grafted seedlings revealed multiple key aspects of this horticultural technique. Frist, the storability of seedlings varied by species and cultivars used for scion and rootstock and therefore grafting nurseries are recommended to examine the storability of grafted seedlings of selected scion and rootstock prior to introducing this technique. Second, while capital infrastructure requires a significant investment, operational electricity costs of storing plants were negligible compared to the total costs for producing grafted plants. Third, the benefit of having the capacity to store grafted seedlings was the reduction of production costs per plant during the peak demand periods. This reduction was attributed to lowering peak production levels, which contributed to reducing the peak labor demand that requires utilizing untrained temporary laborers. In addition, low temperature storage allowed for more seasonal grafters to work for a longer season, increasing their overall grafting speed and productivity. Therefore, low temperature storage can be paid for through improved labor productivity and less uncertainty about being able to satisfy peak demand levels for grafted seedlings. While more specific evaluations need to be done for individual nursery operations, the results shown here are promising for successful introduction of low temperature storage in grafting nurseries.

## Supporting information

S1 File Horticultural analyses visual scores. (XLSX)

S2 File Cost analyses. (XLSX)

S3 File Engineering analyses. (XLSX)
